# Transformation of Malignant Melanoma From Giant Nevus in Infantile Penis

**DOI:** 10.3389/fsurg.2020.594803

**Published:** 2020-12-22

**Authors:** Dong Hwan Lee, Min Ji Kim, Il Jae Lee, Dong Ha Park

**Affiliations:** Department of Plastic and Reconstructive Surgery, Ajou University School of Medicine, Suwon, South Korea

**Keywords:** melanoma, pediatric tumor, penile neoplasms pathology, transformation, pediatric surgery

## Abstract

**Background:** Malignant melanoma is the most serious type of skin cancer, and its incidence rate increases with age. Malignant melanoma in infants has been rarely reported in the literature. Herein, we report a case of malignant transformation of a nodular lesion found in the penis of a patient with a giant congenital nevus.

**Case presentation:** A 1-month-old male patient was admitted due to the presence of a giant congenital nevus involving the lower abdomen, bilateral inguinal areas, genitals, and left thigh and knee. Six months later, nodules measuring 1 cm in diameter protruding from the genital area were noted, and a part of the nodule was removed via elliptical excision with the patient under general anesthesia. Gross examination showed an edematous lesion similar to a neurofibroma and with unclear boundaries. Biopsy revealed a malignant melanoma, with a Breslow thickness of at least 3 mm, and absence of lymphovascular invasion; the biopsy confirmed incomplete excision. The patient was scheduled for radical resection, but reconstruction was not performed following surgical resection due to the guardian's refusal. Hence, the patient only received an adjuvant medical treatment and eventually died.

**Conclusion:** We reported a rare case of an infant with a malignant melanoma in the penis. Congenital malignant melanoma rarely occurs in infants; however, due to its fatal consequences, follow-up should be performed to assess for malignant changes.

## Background

Malignant melanoma is the deadliest form of skin cancer. The incidence rate of malignant melanoma increases with age, and infantile malignant melanoma has rarely been reported in the literature. Moreover, melanoma rarely occurs in individuals aged <20 years (range: 1–4% of all melanoma cases) and is extremely rare among young children. For instance, in infants aged 0–9 months, the annual melanoma incidence rate was found to be 0.7 per million (1, 2).

When estimating the size of a birthmark, a giant congenital nevus is generally defined as a nevus measuring more than 40 cm in diameter (3). It is important to note that birthmarks have a higher risk of becoming malignant, have higher incidence rates of developing hirsutism, and can cause psychiatric problems after adolescence, as well as neurodermal melanoma in rare cases. In particular, a large congenital melanocytic nevus has a 10–15% probability of becoming malignant (4, 5). We present a case of malignant transformation of a nodular lesion identified during follow-up in an infant with a giant congenital nevus.

## Case Report

**Figure 1:**
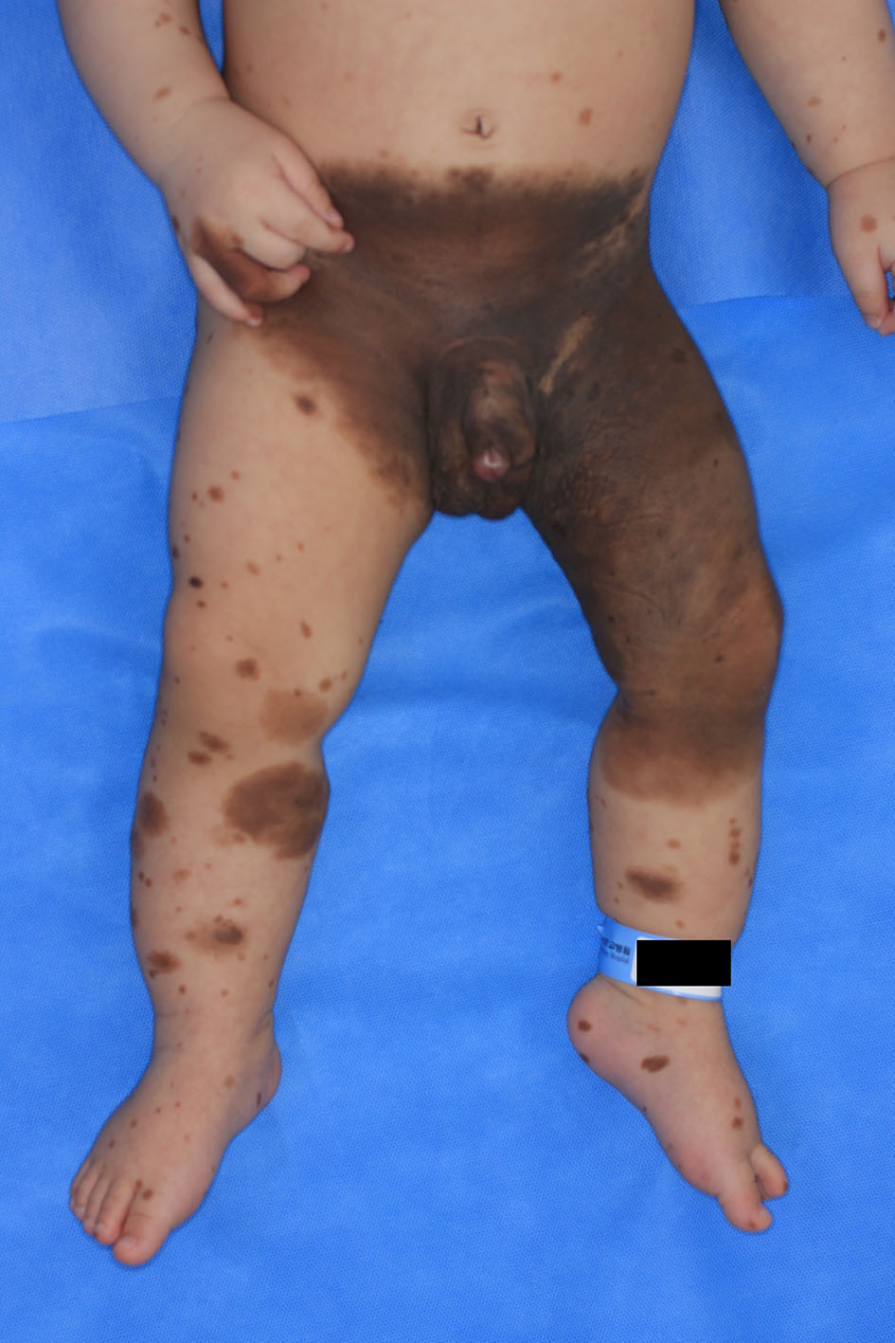
Gross photograph of a congenital giant nevus.

A 1-month-old male infant was brought to Ajou University Hospital due to the presence of a giant congenital nevus encompassing the lower abdomen, right groin, buttocks, penis, left groin, buttocks, thighs, and below the knee with several satellite lesions (Figure 1). He was born at full term, and the pregnancy was uneventful. The patient had no sign of developmental disorders, problems with lactation, systemic disease until 7 months, or family history of cancer and genetic diseases within the second-degree relatives.

**Figure 2:**
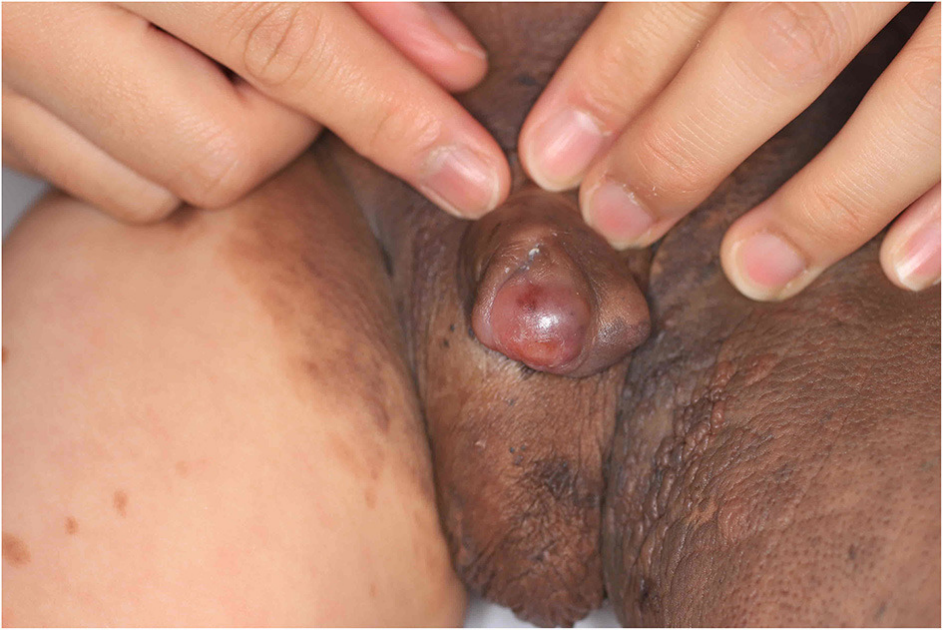
Preoperative photograph of the nodular lesion in the penis.

When the patient first visited the hospital, a partial resection or a step-by-step operation using a tissue expander was planned within a year. The patient's guardian was asked to carefully monitor the nevus for signs of malignant transformation. Six months later, the patient was again brought to the hospital with a nodule measuring 1 cm in diameter bulging in the penis; an excisional biopsy was planned to rule out malignancy (Figure 2).

Results of the gross examination revealed an edematous, soft, and small subcutaneous nodule similar to a neurofibroma mass, with unclear boundaries. The entire nodule could not be removed because of the risk of damaging the functional and anatomical structure; the mass appeared elliptical in shape and measured 3 cm × 1.5 cm.

**Figure 3:**
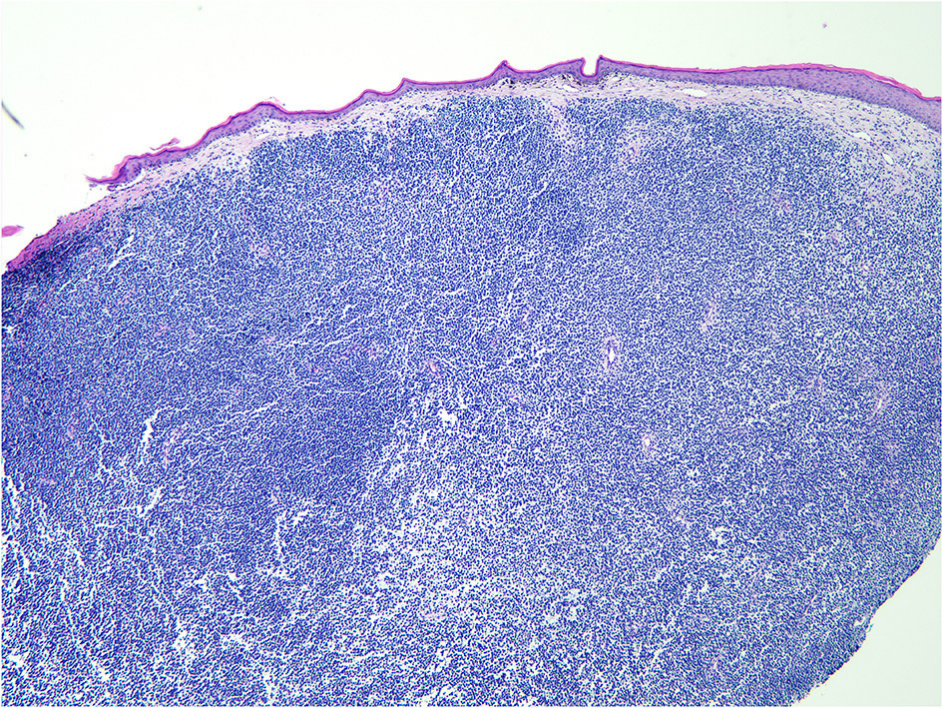
Microscopic pathology of a surgical specimen at 40 × magnification.

**Figure 4:**
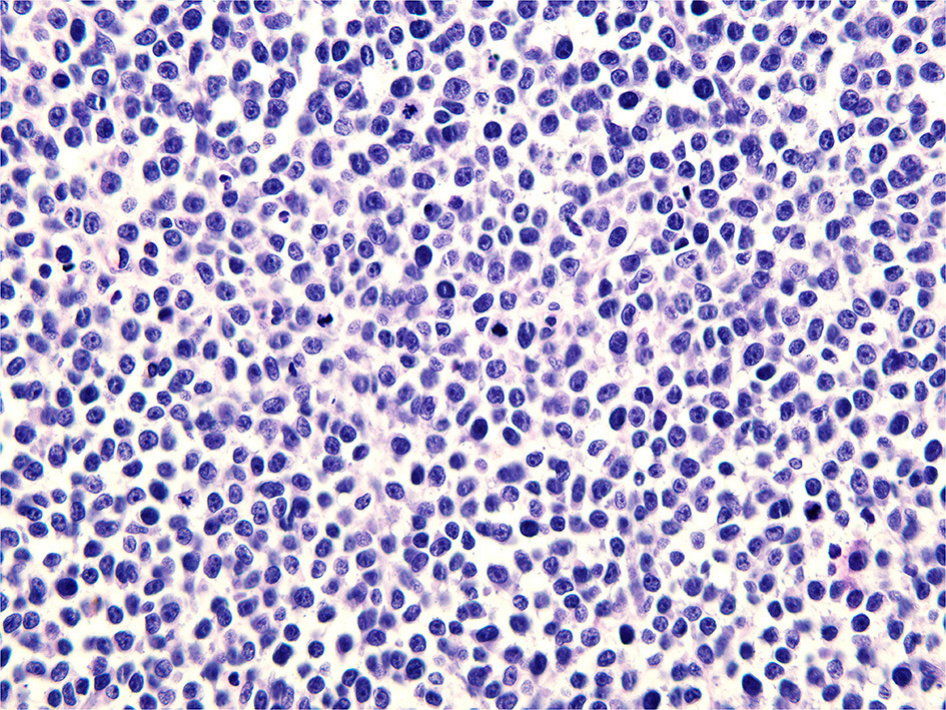
Microscopic pathology of a surgical specimen at 400 × magnification.

A pathological examination was performed on the resected lesion (Figures 3, 4). The brisk mitotic activity was 16/HPF, and the Breslow thickness was at least 3 mm. Lymphovascular invasion was not noted, and tumor-infiltrating lymphocytes were not detected. Immunohistochemical staining revealed the following results: positive for MART-1 and weakly positive for PAX-5. In addition, the patient was negative for CD56, LCA, CK (AE1/AE3), TdT, CD2, CD20, and CD3. The results were incomplete because the procedure was only performed on nodules measuring 1 cm and found on the penis. Primary closure was performed on the wounds and these eventually healed without complications, such as wound opening or infection.

An imaging test to assess for systemic metastasis and a collaborative operation with urology were planned; however, the guardian refused to have the patient undergo radical surgery, and the patient was lost to follow-up. Six months later, the patient underwent a neoadjuvant interferon alpha treatment at another hospital and came back to our institution to ask for a second opinion. The other institution advised that radical surgery, not the interferon alpha treatment, should be implemented first; however, the interferon alpha treatment was started because the guardians refused the surgical treatment. After counseling, the guardian only permitted to continue the interferon alpha treatment, and not proceed with the surgical treatment. Ten months later, when we contacted the guardian to report this case, the patient had already died at the age of 20 months.

## Discussion

Malignant melanoma, which develops in infants, is a rare type of lesion that can occur in three ways: (i) melanoma from the mother that causes distant metastasis through the placenta, (ii) malignant changes in the congenital giant nevus, and (iii) neonatal synthesis of malignant melanoma *in utero*. Richardson et al. analyzed 23 of the 1,925 reported cases of congenital and infantile melanoma in 2002. Of the 22 patients, 10 died (one did not undergo follow-up), and the longest survival time was 18 months (6). In 2004, Leech et al. conducted a literature review of cases of melanoma reported in neonates. Until 2004, nine cases of melanoma originating from congenital birthmarks were reported, and seven showed nodular masses (7).

Giant congenital nevus rarely develops in children and adolescents; however, due to fatal consequences, the nevus should be assessed for malignant changes, and proper follow-up must be performed. Malignant melanoma is difficult to diagnose in children, especially in infants, because its clinical symptoms (color, size, shape, nodularity, ulceration, etc.) and histological findings (mitotic activity, nuclear pleomorphism, etc.) are similar to those of benign tissues. At present, the most accurate method of diagnosing this condition is through the assessment of clinical symptoms and biopsy.

The anatomical location of the primary lesion of a malignant melanoma plays an important role in determining the prognosis. In previous cases, malignant melanomas most commonly developed in the head and neck of infants, with a poor prognosis, especially for the primary lesions in this area (8). The patient in this study developed primary cancer in the penis, but cancer invasion to the genital area was not reported. Melanoma in the urogenital tract rarely develops in individuals at any age: it has been reported in only 0.7% of penile cancer cases and only 0.18% of all melanoma cases (9). We reviewed all case reports on infantile malignant melanoma (Supplementary Table 1). Survival was reported in 23 of 39 patients reported, excluding one who was lost to follow-up. Most results were followed up at the time of the study, and not after a period of 5 years. It is difficult to reflect the actual survival rate. Therefore, it is necessary to follow up with patients for as long as possible in the future.

Wide radical resection is the most important treatment for malignant melanoma in the penis. Depending on the depth and stage, additional treatment may be required. Bilateral inguinocrural dissection may be necessary to determine the possibility of lymph node metastasis. In addition, the administration of chemotherapy or interferon treatment, such as dacarbazine, should be considered. Previous studies showed that treatment with adjuvant interferon alpha significantly reduces the risk of relapse and improves survival (10). Although there is currently a lack of evidence showing the efficacy of this treatment in children, our patient would have shown slightly better prognosis if radical resection was performed and adjuvant interferon alpha treatment was provided. When patients are diagnosed with malignant melanoma, we strongly encourage the guardians to have the patient undergo surgery. Once radical excision of the lesion is performed, other adjuvant treatments can be considered. However, similar to our case, the guardians of very young patients with melanoma are usually reluctant to decide whether a surgery should be performed. It can be hard for even experienced physicians to persuade guardians toward reconstructive surgery when the patient is so young and weak-looking. However, based on our case, once the operation is not performed on time, other adjuvant treatments are no longer beneficial. Our patient eventually died; even though the decision to cancel surgery was made by the patient's guardians, this case is truly heartbreaking. The administration of interferon alpha treatment without prior radical resection was no longer useful.

This case has some limitations. As the patient had already died in another hospital, follow-up was no longer performed and further evaluation, including analysis of the cause of malignant melanoma, could no longer be conducted. Although the exact mechanism of development could not be confirmed, malignant melanoma should be suspected in infants with a giant congenital nevus, considering the potential for distant metastasis during the period of placental and neonatal development *in utero*. In this study, malignant melanoma was diagnosed in this infant through the excisional biopsy of a small nodule in a giant congenital nevus. Malignant melanoma occurs in 1–4% of patients aged below 20 years. However, as shown in this case report, careful observations made by a physician who handled a patient with a giant congenital nevus for the first time confirmed the deformation of the nevus through a follow-up.

In addition, the prognosis of the patient was already poor at the time of analysis as the results were limited due to follow-up loss. Hence, a system must be established so that patients with giant congenital nevus can receive safe and effective treatment with sufficient counseling and mental support when they visit the hospital.

## Conclusion

A large congenital melanocytic nevus has a high risk for malignant transformation, as observed in our patient who was diagnosed with this condition before the age of 1. The finding of this report is meaningful as the case studied is extremely rare and provides indirect experience on their treatment to physicians. Once a malignant melanoma is diagnosed, early radical resection and reconstruction should be considered. There are several obstacles to the surgical treatment of extremely young patients, such as parent's refusal; other adjuvant treatments without prior surgery may not be beneficial. The cancer rapidly progresses and has devastating effects; thus, active surgical treatment should take precedence to reduce the associated mortality.

## Data Availability Statement

The original contributions presented in the study are included in the article/Supplementary Materials, further inquiries can be directed to the corresponding author/s.

## Ethics Statement

The studies involving human participants were reviewed and approved by the institutional review board of Ajou Medical Center (approval no. MED-MDB-20-109). Written informed consent to participate in this study was provided by the participants' legal guardian/next of kin. Informed consent was obtained from the patient's guardian for the publication of any potentially identifiable images or data included in this article.

## Author Contributions

DP conceived and designed this study. DL and IL provided administrative support. DL and MK provided materials and samples and analyzes and interpreted data. DL collected and collated data. All authors read and approved the final manuscript. All authors contributed to the article and approved the submitted version.

## Conflict of Interest

The authors declare that the research was conducted in the absence of any commercial or financial relationships that could be construed as a potential conflict of interest.

## Supplementary Material

Supplementary Table 1 Systematic review of reported cases of congenital or infantile melanoma.
