# Supplementary material for: Transformation of Malignant Melanoma From Giant Nevus in Infantile Penis
Source: Front Surg. 2020 Dec 22;7:594803. doi: 10.3389/fsurg.2020.594803 (PMC7784403; doi:10.3389/fsurg.2020.594803)
Supplement: Supplementary Table 1 — Systematic review of reported cases of congenital or infantile melanoma. [file Table_1.DOCX]

**Supplementary table 1.** Reported cases of congenital or infantile melanoma

| **Case** | **Authors** | **Age at diagnosis** | **Sex** | **Aetiology** | **Location of primary lesion** | **Survival after diagnosis** |
| --- | --- | --- | --- | --- | --- | --- |
| 1 | Coe (1925) | 2–3 months | F | *De novo* | Scalp | D; 5 months |
| 2 | Weber et al. (1930) | 8 months | M | Transplacental | Visceral | D; 10 months |
| 3 | Sweet, Connerty (1941) | 3 h | M | GCMN | Skin | D; 17 days |
| 4 | Dargeon et al. (1950) | 8 months | M | Transplacental | Visceral | D; 11 months |
| 5 | Cavell (1963) | 2 months | F | Transplacental | R leg and thigh | A; time of study |
| 6 | Brodsky et al. (1965) | 48 days | M | Transplacental | Skin | D; 48 days |
| 7 | Lyall (1967) | 11–12 months | M | *De novo* | R 3rd finger | A; 15 years |
| 8 | Oldhoff, Koudstaal (1968) | Birth | M | *De novo* | R thigh | A; 10 years |
| 9 | Conu et al. (1971) | 7 days | F | GCMN | L foot | Lost to follow‐up |
| 10 | Trozak et al. (1975) | 4 months | M | *De novo* | L cheek | A; 20 years |
| 11 | Ahmed (1979) | 2 weeks | M | GCMN | Thigh to chest | D; 4 weeks |
| 12 | Stromberg 1 (1979) | Birth | M | GCMN | R temporal scalp | A; 6 months |
| 13 | Stromberg 2 (1979) | 4 months | M | *De novo* | L mastoid process | A; 18 years |
| 14 | Pratt et al. (1981) | 0–2 years | M | *De novo* | Scalp | A; time of study |
| 15 | Hayes, Green (1984) | Birth | M | *De novo* | Leg | A; 15 years 10 months |
| 16 | Naraysingh(1986) | Birth | M | GCMN | Back | D; 6 weeks |
| 17 | Boddie, Cangir (1987) | 5 months | F | *De novo* | Finger | A; 9 years |
| 18 | Schneiderman et al. (1987) | Birth | M | GCMN | Back | D; ∼ 1 h |
| 19 | Prose et al. (1987) | 6 weeks | F | *De novo* | Mid‐epigastric area | A; 1 year |
| 20 | Song et al. (1990) | Birth | M | GCMN | Posterior neck and scalp | D; 2 h |
| 21 | Ishii et al. (1990) | Birth | M | *De novo* | L thigh | D; 18 months |
| 22 | Broadway et al. (1991) | Birth | F | *De novo* | Eye | A; 2 years 10 months |
| 23 | Baader et al. (1992) | Birth | F | GCMN | Post‐thoracolumbar and gluteal lesion | A; 3 years |
| 24 | Koyama et al. (1996) | Birth | F | GCMN | L parietal scalp | A; time of study |
| 25 | Seigler et al. (1997) | Birth | M | GCMN | Forehead to scalp | A; 4·5 years |
| 26 | Richardson et al. (2002) | 7 months | F | GCMN | Scalp | A; 2 years 5 months |
| 27 | Adedoyin et al. (2004) | 2.5 years | F | *GCMN* | Axilla | A; time of study |
| 28 | Asai et al. (2004) | Birth | M | *De novo* | Thumb | A; 3 years 3 months |
| 29 | Dragieva et al. (2006) | 15 years | F | *GCMN* | Back | D; 16 years |
| 30 | Galinier et al. (2007) | 6 months | M | *GCMN* | Scalp | A; 43 months |
| 31 | Yun et al. (2010) | 2 months | F | *De novo* | Scalp | A; 22 months |
| 32 | Surrenti et al. (2011) | 5 years | M | *GCMN* | Groin | D; 9 years |
| 33 | Feito-Rodriguez et al. (2011) | Birth | F | *GCMN* | Scalp | A; 4 years |
| 34 | Singh et al. (2013) | Birth | M | *De novo* | Scalp | D; 5 months |
| 35 | Katibi et al. (2014) | 3 years | F | *GCMN* | Back | D; 4 years |
| 36 | Su et al. (2014) | Birth | F | *De novo* | Scalp | A; 4 years |
| 37 | Enam et al. (2014) | Birth | M | *De novo* | Scalp | D; 10 months |
| 38 | Volejnikova et al. (2015) | 5 years | M | *GCMN* | Bone marrow | D; 5 years 4 months |
| 39 | Fawcett et al. (2016) | 5 months | M | *De novo* | Generalized disseminated cutaneous lesion | A; 3 years |
| 40 | Maguire et al. (2017) | 7 months | F | *De novo* | Groin | A; time of study |

A: Alive, D: Death, GCMN: Giant congenital melanocytic nevi
